# Supplementary material for: Peroxymonosulfate Activation by Palladium(II) for Pollutants Degradation: A Study on Reaction Mechanism and Molecular Structural Characteristics
Source: Int J Environ Res Public Health. 2022 Oct 11;19(20):13036. doi: 10.3390/ijerph192013036 (PMC9603282; doi:10.3390/ijerph192013036)
Supplement: Supplementary file 1 [file ijerph-19-13036-s001.zip › ijerph-1952540-supplementary.pdf]

Supplementary Materials

Peroxymonosulfate activation by palladium(II) for pollutants degradation: a study on reaction mechanism and molecular structural characteristics

Bowen Yang [1, 2], Qiang Ma [1, 2], Jiming Hao [3] and Xiaojie Sun [4,*]

[1] *Sichuan Provincial Engineering Research Center of City Solid Waste Energy and Buliding Materials Conversion & Utilization Technology, Chengdu University, Chengdu 610106, China.*

[2] *School of Architecture and Civil Engineering, Chengdu University, Chengdu 610106, China.*

[3] *State Key Joint Laboratory of Environment Simulation and Pollution Control, School of Environment, Tsinghua University, Beijing 100084, China.*

[4] *Guangxi Key Laboratory of Environmental Pollution Control Theory and Technology, Guilin University of Technology, Guilin 541004, China.*

** Corresponding author: Xiaojie Sun*

Tel./fax: 15078329789

E-mail: sunxiaojie@glut.edu.cn

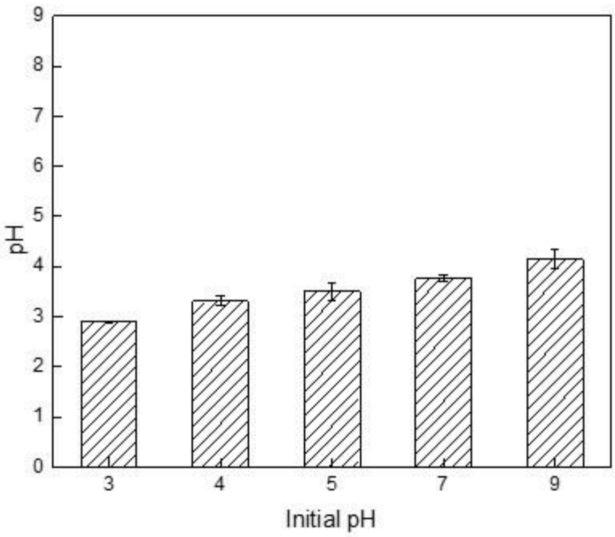

Figure S1. Final solution pH of the experiment regarding the effect of initial pH of 3, 4, 5, 7, and 9 in Pd(II)/PMS system. Condition: [phenol] = 50 μM, [PMS] = 0.25 mM, [Pd(II)] = 10 μM, pH_i = 3, 4, 5, 7, and 9.

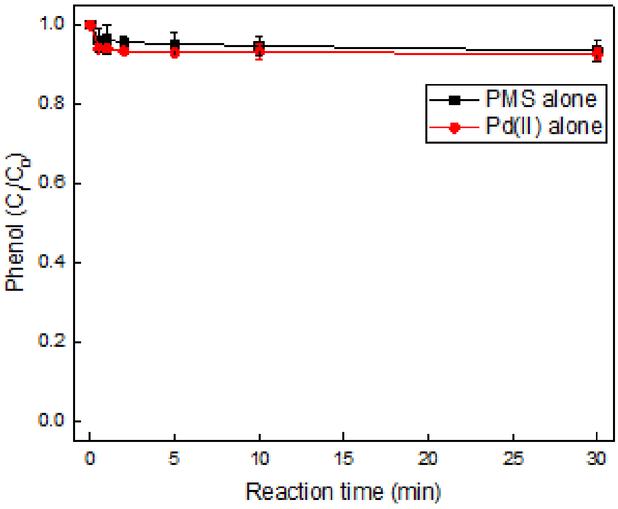

Figure S2. Degradation of phenol by PMS or Pd(II) alone. Condition: [phenol] = 50 μM, [PMS] = 0.25 mM, [Pd(II)] = 10 μM, pH_i = 4.

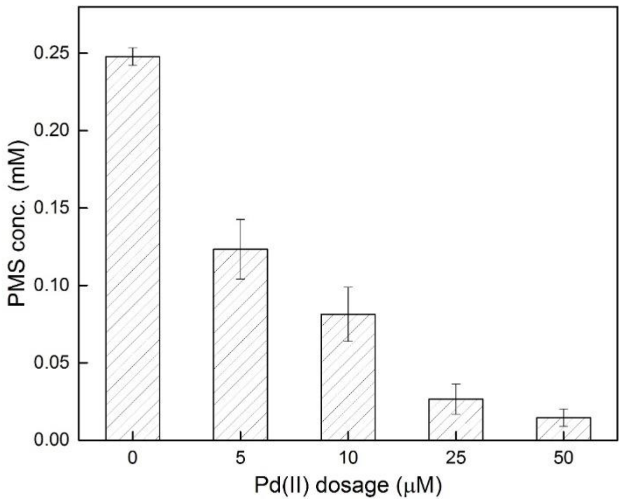

Figure S3. Decomposition of PMS by various Pd(II) dosage after 30 min. Condition: [phenol] = 50 μM, [PMS] = 0.25 mM, [Pd(II)] = 0, 5, 10, 25, and 50 μM, pH_i = 4.

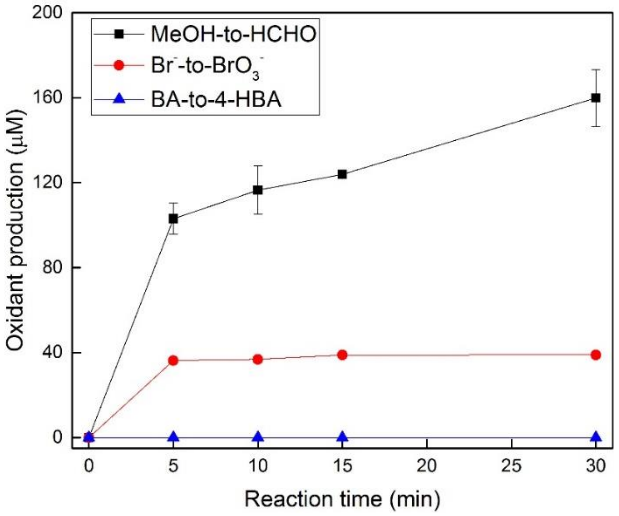

Figure S4. Oxidant production of HCHO, bromate, and 4-HBA in Co(II)/PMS system. Condition: [MeOH] = 200 mM, [Br^-] = 0.1 mM, [BA] = 10 mM, [PMS] = 0.25 mM, [Co(II)] = 25 μM, pH_i = 4.

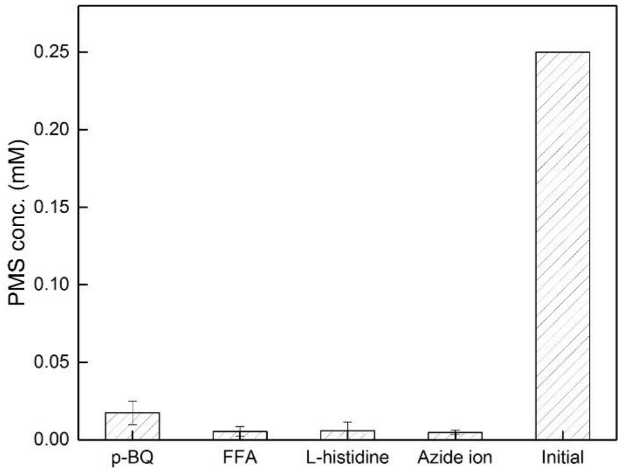

Figure S5. Comparison of PMS concentration between with and without the presence of $^1O_2$ scavengers at the reaction time of 5 min. Condition: [phenol] = 50 μM, [PMS] = 0.25 mM, [Pd(II)] = 25 μM, [FFA] = [L-histidine] = [N_3^-] = 200 mM, [p-BQ] = 100 mM, pH_i = 4.

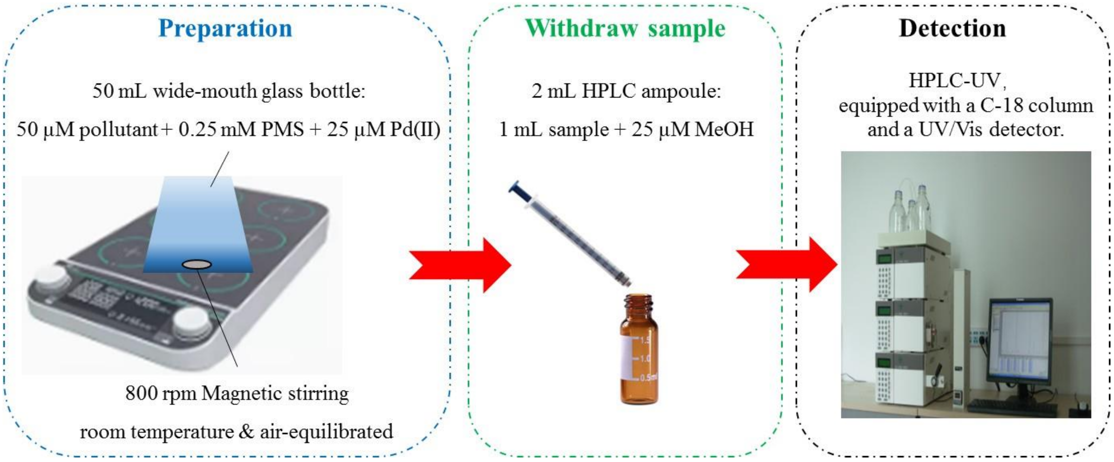

Scheme S1. Typically experimental process in this work from sample preparation to detection by HPLC-UV.

Table S1 Information of chemicals and materials.

Chemical	Abbreviation	CAS
carbamazepine	CBZ	298-46-4
caffeine	Caffeine	58-08-2
bisphenol A	BPA	80-05-7
acid orange 74	AO74	10127-27-2
rhodamine B	RB	81-88-9
1,4-dioxane	1,4-D	123-91-1
4-chlorophenol	4-CP	106-48-9
4-hydroxyphenylacetic acid	4-HBA	156-38-7
4-nitrobenzoic acid	4-NBA	62-23-7
4-nitroaniline	4-NA	100-01-6
4-nitrophenol	NP	100-02-7
benzoic acid	BA	65-85-0
nitrobenzene	NB	98-95-3
phenol	Phenol	108-95-2
2,4-dichlorophenol	DCP	120-83-2
2,4,6-trichlorophenol	TCP	88-06-2
p-benzoquinone	p-BQ	106-51-4
sodium azide	NaN_3	26628-22-8
L-histidine	L-histidine	71-00-1
Oxone	PMS	10058-23-8
palladium(II) chloride	Na_2PdCl_4	7647-10-1
cobalt(II) chloride	$CoCl_2$	7646-79-9
sodium hydroxide	NaOH	8012-01-9
perchloric acid	$HClO_4$	7601-90-3
sodium bromide	NaBr	7647-15-6
sodium bromate	$NaBrO_3$	7789-38-0
potassium iodide	KI	7681-11-0
phosphoric acid	H_3PO_4	7664-38-2
5-tert-butoxycarbonyl-5-methyl-1-pyrroline-*N*-oxide	BMPO	387334-31-8
Deuterium oxide	D_2O	7789-20-0
2,4-dinitrophenylhydrazine	DNPH	119-26-6
methanol	MeOH	67-56-1
tert-butyl alcohol	TBA	75-65-0
furfuryl alcohol	FFA	98-00-0
acetonitrile	ACN	75-05-8
formaldehyde	HCHO	50-00-0

Ultrapure water (> 18 MΩ•cm) produced by a Milli-Q Water Purifcation System (Millipore) was used for the preparation of all experimental solutions and suspensions.

Table S2 Operating conditions for high-performance liquid chromatographic analysis.

Compound	Tube A (%)	Tube B (%)	Tube C (%)	Wavelength (nm)	Flowrate (mL min^{-1})
phenol	50	50	0	277	0.8
CBZ	60	30	10	215	1.0
Caffeine	60	0	40	264	0.8
BPA	55	45	0	230	0.8
RB	25	30	45	546	0.5
AO74	50	25	25	476	0.6
1,4-D	95	5	0	190	1.0
BA	50	50	0	227	0.8
4-HBA	80	20	0	270	0.8
NB	40	0	60	275	0.8
NP	60	20	20	317	0.8
4-NBA	50	50	0	270	0.5
4-NA	60	0	40	254	1.0
4-CP	60	40	0	230	0.8
DCP	30	70	0	280	1.0
TCP	30	0	70	290	1.0
HCHO	50	50	0	350	1.0

Tube A, B, and C stood for 0.1% H_3PO_4, ACN, and MeOH, respectively.

Table S3 The interpretation on 17 molecular characters.

Characters	Interpretation
μ	Dipole moment
E_{B3LYP}	The total energy of a molecule
E_{HOMO}	Energy of the highest occupied molecular orbital
E_{LUMO}	Energy of the lowest unoccupied molecular orbital
$q(H)_x$	Most positive partial charge on a hydrogen atom
$q(C)_n/q(C)_x$	Minimum and maximum negative partial charge on a carbon atom
$q(C\text{-}H)_n/q(C\text{-}H)_x$	Minimum and maximum positive partial charge on a hydrogen atom linked with a carbon atom
BO_n/BO_x	Minimum and maximum number of chemical bonds between a pair of coterminous atoms
$F(0)_n/F(0)_x$	Minimum and maximum value of Fukui indices by hydroxyl radical attack
$F(+)_n/F(+)_x$	Minimum and maximum value of Fukui indices by nucleophilic attack
$F(-)_n/F(-)_x$	Minimum and maximum value of Fukui indices by electrophilic attack

Table S4 Relationship between degradation rate (*k*) and 17 molecular descriptors of 16 target pollutants in Pd(II)/PMS system.

Compound	*k* (min^{-1})	μ (Debye)	E_{B3LYP} (eV)	E_{HOMO} (eV)	E_{LUMO} (eV)	$q(H)_x$ (e)	$q(C)_n$ (e)	$q(C)_x$ (e)	$q(C\text{-}H)_n$ (e)	$q(C\text{-}H)_x$ (e)	BO_n	BO_x	$F(-)_n$ (e)	$F(-)_x$ (e)	$F(+)_n$ (e)	$F(+)_x$ (e)	$F(0)_n$ (e)	$F(0)_x$ (e)
phenol	0.589	1.583	-307	-0.164	0.009	0.460	-0.254	0.347	0.185	0.208	0.879	1.387	0.040	0.111	0.032	0.102	0.055	0.095
Caffeine	0.057	4.694	-681	-5.834	-0.988	0.404	-0.365	0.837	0.164	0.243	0.885	1.812	0.012	0.106	0.015	0.125	0.016	0.106
CBZ	0.232	3.555	-763	-6.041	-1.614	0.389	-0.200	0.838	0.200	0.218	0.920	1.813	0.007	0.079	-0.002	0.089	0.006	0.077
BPA	0.314	1.583	-653	-0.043	0.011	0.465	-0.312	0.330	0.158	0.212	0.878	1.373	-0.026	0.077	-0.016	0.061	-0.019	0.056
RB	0.424	9.205	-1881	-0.229	-0.012	0.382	-0.581	0.438	0.190	0.280	0.962	1.530	-0.007	0.038	-0.005	0.063	-0.006	0.062
AO74	0.363	13.83	-1597	-0.279	-0.016	0.484	-0.296	0.477	0.166	0.226	1.045	1.679	-0.012	0.033	0.014	0.034	0.012	0.033
1,4-D	0.513	0	-307	-6.607	1.333	0.183	-0.050	-0.050	0.155	0.183	0.874	0.978	0.052	0.174	0.057	0.100	0.057	0.118
BA	0.059	1.243	-217	-0.088	0.385	0.206	-0.588	0.039	0.189	0.206	0.964	1.381	0.044	0.101	-0.023	0.096	-0.022	0.096
4-HBA	0.141	1.868	-496	-6.656	-1.284	0.479	-0.291	0.362	0.202	0.225	0.933	1.864	0.031	0.132	0.033	0.131	0.036	0.109
NB	0.052	5.145	-437	-0.276	-0.140	0.338	-0.291	0.061	0.208	0.238	1.323	1.590	-0.022	0.060	0.035	0.094	0.016	0.077
NP	0.084	5.304	-512	-0.262	-0.089	0.468	-0.282	0.366	0.207	0.239	1.296	1.836	0.007	0.087	0.045	0.116	0.066	0.096
4-NBA	0.065	3.672	-930	-0.320	-0.128	0.489	-0.157	0.815	0.262	0.276	0.909	1.913	0.010	0.224	0.019	0.121	0.002	0.141
4-NA	0.125	7.846	-1203	-0.224	-0.119	0.469	-0.234	0.762	0.210	0.255	0.778	1.642	-0.015	0.233	-0.006	0.143	-0.003	0.135
4-CP	0.786	2.544	-767	-6.337	-0.751	0.463	-0.273	0.335	0.203	0.219	0.955	1.389	0.039	0.220	0.029	0.123	0.037	0.166
DCP	1.220	3.148	-1226	-6.563	-1.067	0.468	-0.255	0.307	0.207	0.235	0.951	1.381	0.032	0.196	0.027	0.142	0.035	0.151
TCP	1.675	1.384	-1686	-6.814	-1.369	0.480	-0.248	0.278	0.238	0.238	0.919	1.333	0.032	0.191	0.022	0.138	0.030	0.145
**Correlation coefficient**		**-0.242**	**-0.467**	**-0.509**	**-0.318**	**0.228**	**0.180**	**-0.265**	**-0.203**	**-0.101**	**-0.209**	**-0.543**	**0.408**	**0.377**	**0.210**	**0.275**	**0.315**	**0.447**
